# How does the multidimensional frailty score compare with grip strength for predicting outcomes after hip fracture surgery in older patients? A retrospective cohort study

**DOI:** 10.1186/s12877-021-02150-9

**Published:** 2021-04-07

**Authors:** Jung-Yeon Choi, Jin-Kak Kim, Kwang-il Kim, Young-Kyun Lee, Kyung-Hoi Koo, Cheol-Ho Kim

**Affiliations:** 1 Department of Internal Medicine, Seoul National University Bundang Hospital, 82, Gumi-ro 173beon-gil, Bundang-gu, Seongnam-si, Gyeonggi-do Republic of Korea; 2 Department of Orthopedic Surgery, Seoul National University Bundang Hospital, 82, Gumi-ro 173beon-gil, Bundang-gu, Seongnam-si, Gyeonggi-do Republic of Korea; 3 Department of Internal Medicine, Seoul National University College of Medicine, 103, Daehak-ro, Jongno-gu, Seoul, Republic of Korea; 4 Department of Orthopedic Surgery, Seoul National University College of Medicine, 103, Daehak-ro, Jongno-gu, Seoul, Republic of Korea

**Keywords:** Frailty, Grip strength, Hip fracture, Multidimensional frailty score, Postoperative complication

## Abstract

**Background:**

Frailty and low handgrip strength (HGS) are associated with adverse outcomes after hip fracture (HF) surgery. We aimed to compare the predictive role of frailty and HGS for adverse outcome in HF patients.

**Methods:**

We included older patients (age ≥ 65 years) who underwent HF surgery to compare the predictive role of HGS and hip-multidimensional frailty score (Hip-MFS) for postoperative complications and mortality. The Hip-MFS was calculated based on comprehensive geriatric assessment (CGA), and HGS was measured with a hand dynamometer. The primary outcome was a composite of postoperative complications (e.g., pneumonia, urinary tract infection, delirium, acute pulmonary thromboembolism, and unplanned intensive care unit admission). The secondary outcomes were 6-month mortality and mortality at the end of follow-up.

**Results:**

The median observation time was 620.5 days (interquartile range: 367.0–784.8 days). Among the 242 patients (mean age: 81.5 ± 6.7 years, 73.1% women), 106 (43.8%) experienced postoperative complications. The 6-month mortality and mortality at the end of follow-up were 7.4% (*n* = 18) and 20.7% (*n* = 50), respectively. The Hip-MFS (odds ratio [OR], 1.250; 95% confidence interval [CI], 1.092–1.432) and HGS (OR, 1.147; 95% CI, 1.082–1.215) could predict postoperative complications. The Hip-MFS could predict both 6-month mortality (hazard ratio [HR], 1.403; 95% CI, 1.027–1.917) and mortality at the end of follow-up (HR, 1.493; 95% CI, 1.249–1.769) after adjustment, while HGS was only associated with mortality at the end of follow-up (HR, 1.080; 95% CI, 1.024–1.139). For mortality at the end of follow-up, predictive models with the Hip-MFS were superior to those with HGS in the time-dependent receiver-operating curve analysis after adjustment (*p* = 0.017). Furthermore, the addition of Hip-MFS or HGS to the American Society of Anesthesiologists (ASA) classification improved its prognostic ability.

**Conclusions:**

Both the Hip-MFS and HGS could predict postoperative complications and improve prognostic utility when combined with the ASA classification. The Hip-MFS was a stronger predictor of mortality than HGS after HF surgery. HGS could be a useful pre-screening tool to identify patients at a high risk of postoperative complications and those who may benefit from further CGA.

**Supplementary Information:**

The online version contains supplementary material available at 10.1186/s12877-021-02150-9.

## Background

Hip fracture (HF) is a catastrophic fracture that leads to considerable morbidity, loss of independence, increased health care cost, and 24% of mortality within 1 year. The incidence of HF increased by 1.23-fold from 2006 to 2015 in Korea with aging of the population [1–3]. As most of the morbidity or mortality cases are due to general medical conditions and not fracture itself, risk stratification for patients at risk of adverse outcomes and selection of candidates for interventions are important to reduce risk [4, 5].

Handgrip strength (HGS) and gait speed are known to predict further morbidity or mortality in older adults [4, 6, 7]. In particular, HGS can be easily assessed in bed-bound HF patients. HGS is considered to be an index of muscle strength and is known to predict HF outcomes [6]. Frailty is a clinical state of increased vulnerability associated with morbidity and mortality when exposed to a stressor, especially after surgery [8]. Frailty is an important geriatric syndrome caused by multiple contributors characterized by diminished muscle strength, endurance, and reduced function [9].

Previous studies have reported various predictors of adverse clinical outcomes for older HF patients. A recent systematic review reported that comorbidity, sarcopenia, surgical factors, socio-economic status, systemic factors, and two emerging predictors, HGS and frailty, could influence outcomes after HF [10]. The hip-multidimensional frailty score (Hip-MFS) is calculated from comprehensive geriatric assessment (CGA) and uses the concept of cumulative deficit to assess frailty. The Hip-MFS was developed and validated to predict adverse outcomes and short-term mortality more precisely than chronological age or conventional risk stratification tools after HF surgery [11]. Given that the CGA is a costly, time-consuming, and labor-intensive process, simple physical performance parameters such as HGS are considered as reliable indicators for the rapid assessment of preoperative risk [6, 12]. Although HGS has been validated to predict adverse events, no previous study has compared the predictive utility of HGS with the Hip-MFS after HF surgery.

Thus, we aimed to compare the prognostic value of the composite frailty score (Hip-MFS), HGS, and other conventional risk factors (American Society of Anesthesiologists [ASA] Physical Status Classification System) in predicting the occurrence of postoperative complications or mortality in older patients who have undergone HF surgery [13]. Furthermore, we examined the additive effect of the Hip-MFS and HGS on the predictive value of the conventional pre-anesthesia assessment, the ASA classification, for the prognostic assessment of postoperative complications.

## Methods

### Study design and setting

This research was conducted in one 1300-bed teaching tertiary hospital between January 1, 2016, and June 30, 2018 with retrospective cohort design. The study protocol was reviewed and approved by the institutional review board of Seoul National University Bundang Hospital, which waived the requirement for informed consent because it is practically impossible to obtain consent from each participant and even if the consent is exempted, the risk to the participant is extremely low [B-1911/579–104].

### Participants

Older (age ≥ 65 years) HF patients who were referred to a geriatric team for pre-surgical CGA were included. If the HF surgery was performed more than twice during the period, only the data corresponding to the first operation were included. Patients with missing HGS data or components of the Hip-MFS data were excluded. Baseline demographic, anthropometric, laboratory, and ASA classification data of the included patients were retrieved from the electronic medical record systems.

### Assessment

HGS (kg) was assessed in the dominant hand in the supine position, with their elbows fixed at 90° using the Jamar Plus+ Digital Hand Dynamometer (Sammons Preston, Bolingbrook, IL, USA). The maximum HGS among two measurements was used for analysis. The cut-off value for low HGS was defined as the sex-specific quintile point (lowest 20%, < 28.6 kg and < 16.4 kg in men and women, respectively) obtained from a healthy older population in the Korea National Health and Nutrition Examination Survey IV [14]. The Hip-MFS was calculated from the CGA (comorbidity, cognitive function, walking ability, risk of falling, nutritional status), demographic (sex), and laboratory test data (serum albumin), with a range of 0 to 14 [11]. A detailed description of the practical assessment and the cut-off values have been presented previously. A high Hip-MFS score was identified as a score of more than 8 (Hip-MFS > 8), as adopted in previous study [11].

The CGA is multidisciplinary diagnostic process which is now the accepted gold standard for caring for older hospitalized patients. The CGA was used to evaluate the following six sub-domains: comorbidity, medications, physical function, psychosocial function, nutritional status, and risk of delirium. Comorbid conditions were evaluated using the Charlson Comorbidity Index, and the Modified Barthel Index, Lawton & Brody Index, and Koval grade were used to assess patient’s activities of daily living (ADLs), instrumental ADLs (IADLs), and walking ability, respectively. The psychosocial function was determined by evaluating the patients’ cognitive function and depressive status using the Korean version of the Mini-Mental State Examination (MMSE-KC) and the Korea Geriatric Depression Scale (SGDS-K). The validated nutrition screening tool, the Mini Nutritional Assessment (MNA), was used to assess patients who were malnourished or at risk of malnutrition. The risk of delirium was evaluated using the Nursing Delirium Screening Scale. The risk of falling was evaluated using the Predisposition for Falling assessment guide, as most cases of low traumatic HF are caused by falls [15].

### Outcome

The primary outcome was a composite outcome of the following postoperative complications: delirium, pneumonia, urinary tract infection, pulmonary thromboembolism, and unplanned intensive care unit (ICU) admission after surgery. Delirium was identified by psychiatric consultation with a state of acute confusion or by retrospective chart review, and based on the Diagnostic and Statistical Manual of Mental Disorder, 5th edition, criteria [16]. Defined criteria of the National Surgical Quality Improvement Program were used to identify each condition of pneumonia, urinary tract infection, and pulmonary thromboembolism [17]. Unplanned ICU admission was defined as transfer from general ward to an ICU within a hospitalization period of at least 72 h after HF surgery for close monitoring for any medical or surgical issues. The secondary outcomes were 6-month mortality and mortality at the end of follow-up, which were assessed using the data of National Statistical Office from January 1, 2016, until December 31, 2018. The methodology of the CGA and definition of study outcome was defined in the same way as the previous studies conducted in SNUBH [11, 18].

### Statistical analysis

The data were analyzed using IBM SPSS Statistics for Windows, version 25.0 (IBM Corp., Armonk, NY, USA), Stata SE, version 15 (StataCorp, College Station, TX, USA), R version 3.4.4 (The R Foundation for Statistical Computing, Vienna, Austria), and MedCalc Statistical Software version 17.5.3 (MedCalc Software, Ostend, Belgium). Continuous variables are expressed as mean (standard deviation [SD]) or median (interquartile range [IQR]) and were compared using t-test. Discrete variables are presented as numbers or percentages, and the chi-square or Fisher’s exact test was used to compare proportions. Effects of the Hip-MFS and HGS on primary or secondary outcomes were analyzed by multivariate logistic regression models or Cox’s proportional hazard analysis, respectively. We compared the predictive value of the Hip-MFS for the primary outcome with that of the ASA classification and HGS using a receiver-operating characteristic (ROC) curve. The additional prognostic utility of the Hip-MFS and HGS when added to the ASA classification was evaluated by comparing the C-index, which measures the goodness of fit in a logistic regression model. Model 1 was adjusted for age, sex, and body mass index (BMI). Model 2 was adjusted for Model 1 and the type of fracture (neck or intertrochanteric fracture). Model 3, the final model, was adjusted for Model 2 and various laboratory variables (white blood cell count, hemoglobin, total cholesterol, protein, blood urea nitrogen, and creatinine). The Kaplan–Meier analysis was used to analyze the survival curves, and log-rank tests were used to assess the significance between the risk groups. Time-dependent ROC curve analysis based on 1000 days was performed to assess the predictive performance of the Hip-MFS and HGS, and DeLong’s method was used to compare between time-dependent ROC curve of Hip-MFS and HGS. All statistical analyses were two-tailed, and *p*-values < 0.05 were considered statistically significant.

## Results

During the study period, 455 patients underwent HF surgery, among whom, 274 (60.2%) underwent CGA. After excluding 30 patients with missing data on HGS, 1 who developed aspiration pneumonia before HF surgery, and 1 who had missing data required to calculate the Hip-MFS, 242 patients were included in the final analysis.

The mean age of study participants was 81.5 years (SD, 6.7), and 73.1% (*n* = 177) were women. A total of 106 (43.8%) patients experienced postoperative complications, including pneumonia (14 patients), urinary tract infection (9 patients), delirium (100 patients), stroke (1 patient), and unplanned ICU admission (11 patients). Fifteen and seven patients experienced two and three simultaneous postoperative complications, respectively. The median observation time was 620.5 days (IQR: 367.0–784.8). The mortality at the end of follow-up was 20.7% (*n* = 50), while the 6-month mortality after HF surgery was 7.4% (*n* = 18).

Older patients and those with lower BMI, higher ASA classification, lower serum cholesterol, lower protein, and lower albumin levels tended to experience more postoperative complications. Among the CGA domains, dependent ADL, IADL, lower MMSE-KC, MNA score, higher SGDS-K score, and positive risk of falling were related to complications. Weaker HGS strength and a higher Hip-MFS score were also correlated with complications **(**Table 1**).**

**Table 1:** Comparison of demographic, laboratory, and CGA domains by postoperative complication

	No Complication (***n*** = 136)	Complication (***n*** = 106)	***p***-values
**Demographic**			
Age (year)	80.3 (6.4)	83.1 (6.8)	***0.001***
Sex (male/female)	35/101	30/76	0.655
Body mass index (kg/m^2^)	22.0 (3.7)	20.9 (3.8)	***0.016***
ASA class (1/2/3/4) ^a^	6/77/29/3	6/43/43/1	***0.050***
Type of fracture (neck/intertrochanter)	66/70	55/51	0.604
Anesthesia (general/spinal)	109/27	94/12	0.073
**Laboratory**			
WBCs (× 10^3^/μL)	9.8 (3.6)	9.7 (3.5)	0.828
Hemoglobin (g/dL)	11.5 (2.0)	11.1 (2.0)	0.093
Platelets (× 10^3^/μL)	216.1 (81.0)	198.2 (73.0)	0.076
BUN (mg/dL)	20.0 (11.9)	22.3 (14.3)	0.165
Creatinine (mg/dL)	0.99 (0.92)	1.22 (1.44)	0.137
Total Cholesterol (mg/dL)	162.0 (37.8)	150.4 (35.5)	***0.016***
Protein (mg/dL)	6.7 (0.6)	6.5 (0.7)	***0.024***
Albumin (mg/dL)	3.8 (0.4)	3.5 (0.5)	***< 0.001***
**Comprehensive geriatric assessment**			
Charlson’s comorbidity index	1.5 (1.7)	1.9 (1.8)	0.118
Polypharmacy (≥5)	96 (70.6%)	78 (73.6%)	0.607
ADL dependency (partial and full) ^b^	58 (42.6%)	69 (65.7%)	***< 0.001***
IADL dependency ^b^	70 (51.5%)	80 (76.2%)	***< 0.001***
MMSE-KC	20.4 (6.4)	14.8 (7.1)	***< 0.001***
SGDS-K^c^	4.9 (3.7)	6.4 (4.6)	***0.025***
Risk of falling (≥10)	42 (30.9%)	55 (51.9%)	***0.001***
MNA	22.0 (4.2)	19.6 (4.7)	***< 0.001***
Mid-arm circumference (cm)	24.4 (11.4)	22.6 (3.1)	0.112
Grip strength (kg)	17.7 (7.4)	13.4 (5.9)	***< 0.001***
The Koval grade	2.2 (1.7)	2.6 (1.8)	0.074
Hip-MFS	6.1 (2.8)	7.8 (2.3)	***< 0.001***

Data are presented as mean (SD) or number (%)

*ADL* activities of daily living, *ALT* alanine aminotransferase, *AST* aspartate aminotransferase, *ASA* American Society of Anesthesiologists, *CGA* comprehensive geriatric assessment, *IADL* instrumental activities of daily living, *MMSE-KC* Korean version of the mini-mental status examination, *SGDS-K* short form of the Korean Geriatric Depression Scale, *MNA* Mini Nutritional Assessment, *Nu-DESC* Nursing Delirium Screening Scale, *WBC* white blood cell

^a^Data were missing for 34 patients

^b^Data were missing for 1 patient

^c^Data were missing for 64 patients

Both the Hip-MFS and HGS, but not the ASA classification, could predict postoperative complications in the fully adjusted model (Model 3), with odds ratios (ORs) of the Hip-MFS (per point), HGS (per kg), and ASA classification of 1.250 (95% confidence interval [CI]: 1.092–1.432, *p* = 0.001), 1.147 (95% CI 1.082–1.215, *p* <  0.001), and 1.347 (95% CI: 0.804–2.257, *p* = 0.258), respectively **(**Table 2**).**

**Table 2:** Adjusted odds ratios and hazard ratios by grip strength, gait speed, and other risk factors for postoperative complications, 6-month mortality, and mortality at the end of follow-up

	Model 1^**a**^	Model 2^**b**^	Model 3^**c**^
Complication			
Grip strength (per kg)	1.147 (1.085–1.213)^†^	1.151 (1.088–1.218)^†^	1.147 (1.082–1.215)^†^
Hip-MFS	1.260 (1.118–1.422)^†^	1.275 (1.127–1.442)^†^	1.250 (1.092–1.432)*
ASA classification^d^	1.566 (0.971–2.526)	1.563 (0.969–2.521)	1.347 (0.804–2.257)
6-month mortality			
Grip strength (per kg)	1.116 (1.023–1.217)^*^	1.111 (1.015–1.216)^*^	1.101 (0.985–1.231)
Hip-MFS	1.603 (1.235–2.080)^‡^	1.530 (1.183–1.979)^†^	1.403 (1.027–1.917)^*^
ASA classification^d^	2.236 (1.076–4.645)^*^	2.449 (1.134–5.290)^*^	3.066 (1.106–8.503)^*^
Mortality at the end of follow-up			
Grip strength (per kg)	1.094 (1.040–1.152)^∥^	1.093 (1.037–1.151)^†^	1.080 (1.024–1.139)^*^
Hip-MFS	1.546 (1.334–1.795)^‡^	1.538 (1.323–1.788)^‡^	1.493 (1.259–1.769)^*^
ASA classification ^d^	1.725 (1.094–2.718)^*^	1.750 (1.106–2.770)^*^	1.551 (0.908–2.651)

Data are presented as hazard ratio (95% confidence interval)

^a^Adjusted by age, sex, and body mass index

^b^Adjusted by age, sex, body mass index, and type of fracture

^c^Adjusted by age, sex, body mass index, type of fracture, white blood cell count, hemoglobin, total cholesterol, protein, blood urea nitrogen, and creatinine

^d^Data were missing for 34 patients

^*^p < 0.05, ^†^*p* < 0.01, ^‡^*p* < 0.001

**Fig. 1:**
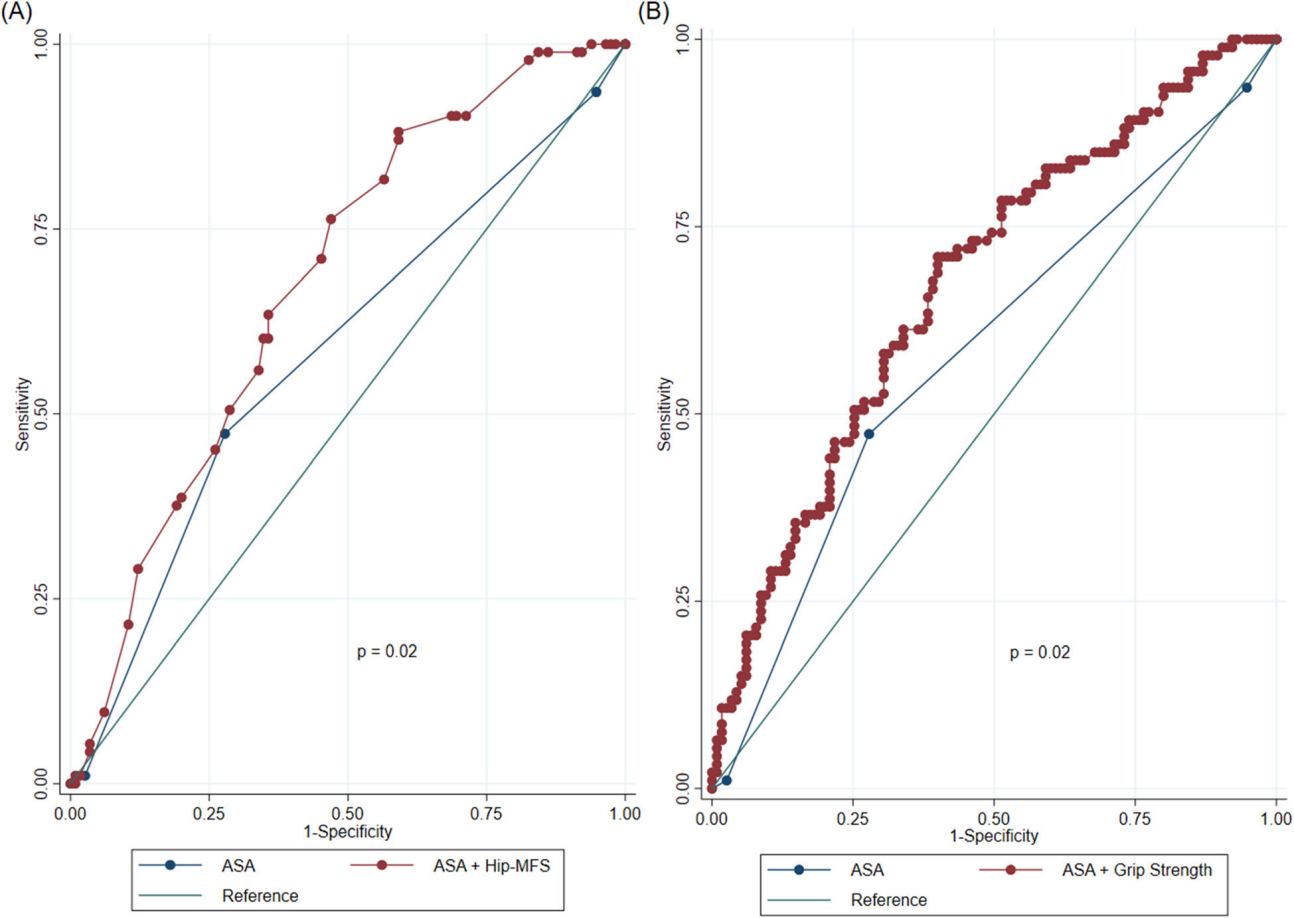
Additional prognostic utility of the Hip-MFS and grip strength with the ASA classification in predicting postoperative complications. **a** ASA vs. ASA + Hip-MFS. **b** ASA vs. ASA + grip strength. Hip-MFS: hip-multidimensional frailty score, ASA: American Society of Anesthesiologists

The Hip-MFS was more accurate than the ASA classification in predicting postoperative complications according to the area under the curve (AUC), with a *p*-value for analyzing the difference between the AUC of the Hip-MFS (AUC: 0.679; 95% CI: 0.613–0.745) and the AUC of the ASA classification (AUC: 0.583; 95% CI: 0.513–0.653) of 0.037. However, the Hip-MFS was not significantly superior to HGS in predicting postoperative complications (AUC: 0.669, 95% CI: 0.602–0.737, *p* = 0.793). Moreover, the AUCs of HGS and ASA classification were not significantly different (*p* = 0.080). Compared with the C-index of the logistic model of ASA classification alone or in combination with the Hip-MFS or HGS, the predictive value of the ASA + Hip-MFS (C-index: 0.679, 95% CI: 0.607–0.751) or ASA + HGS (C-index: 0.676, 95% CI: 0.603–0.749) was superior to that of the ASA classification alone (C-index 0.583, 95% CI 0.513–0.653) for predicting postoperative complications (*p* = 0.0198 and 0.0204, respectively) (Fig. 1).

**Fig. 2:**
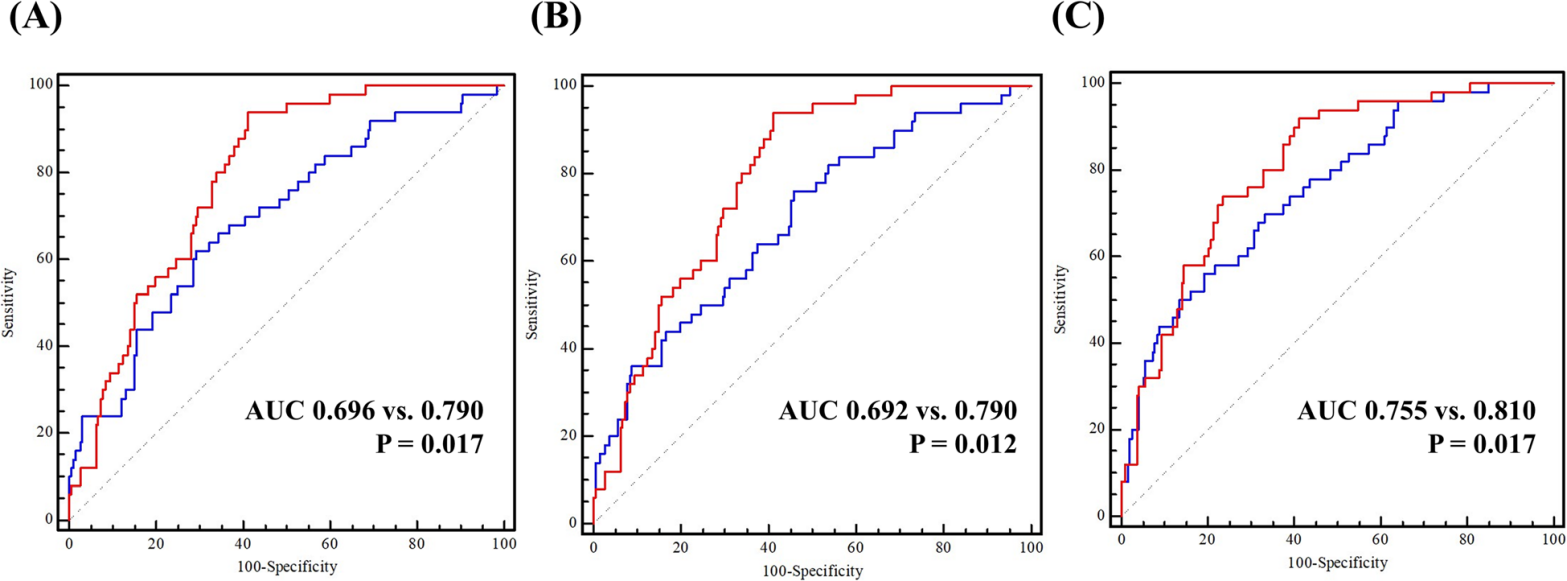
Time-dependent receiver-operating characteristic (ROC) curves at 1000 days based on the Hip-MFS- (red) and grip strength- (blue) based prognostic model for mortality at the end of follow-up. **a** Model 1: Adjusted by age, sex, and body mass index. **b** Model 2: Adjusted by age, sex, body mass index, and type of fracture. **c** Model 3: Adjusted by age, sex, body mass index, type of fracture, white blood cell count, hemoglobin, total cholesterol, protein, blood urea nitrogen, and creatinine. Hip-MFS: hip-multidimensional frailty score

The hazard ratio (HR) in the fully adjusted (Model 3) model of the Hip-MFS for predicting 6-month mortality was 1.403. (95% CI: 1.027–1.917, *p* = 0.033). The ASA classification could also predict the 6-month all-cause mortality (HR: 3.066, 95% CI: 1.106–8.503, *p* = 0.031), whereas HGS could not (HR: 1.101, 95% CI: 0.985–1.231, *p* = 0.089). Furthermore, the Hip-MFS (HR: 1.493, 95% CI: 1.259–1.769, *p* <  0.001) and HGS (HR: 1.080, 95% CI: 1.024–1.139, *p* = 0.005), but not the ASA classification (HR: 1.551, 95% CI: 0.908–2.651, *p* = 0.108) could predict mortality at the end of follow-up in the fully adjusted model (Model 3) **(**Table 2**).** Furthermore, with regard to predicting mortality at the end of follow-up, predictive models with the Hip-MFS were superior to those with HGS, with *p*-values of 0.017, 0.012, and 0.017 in models 1, 2, and 3, respectively **(**Fig. 2**).**

**Fig. 3:**
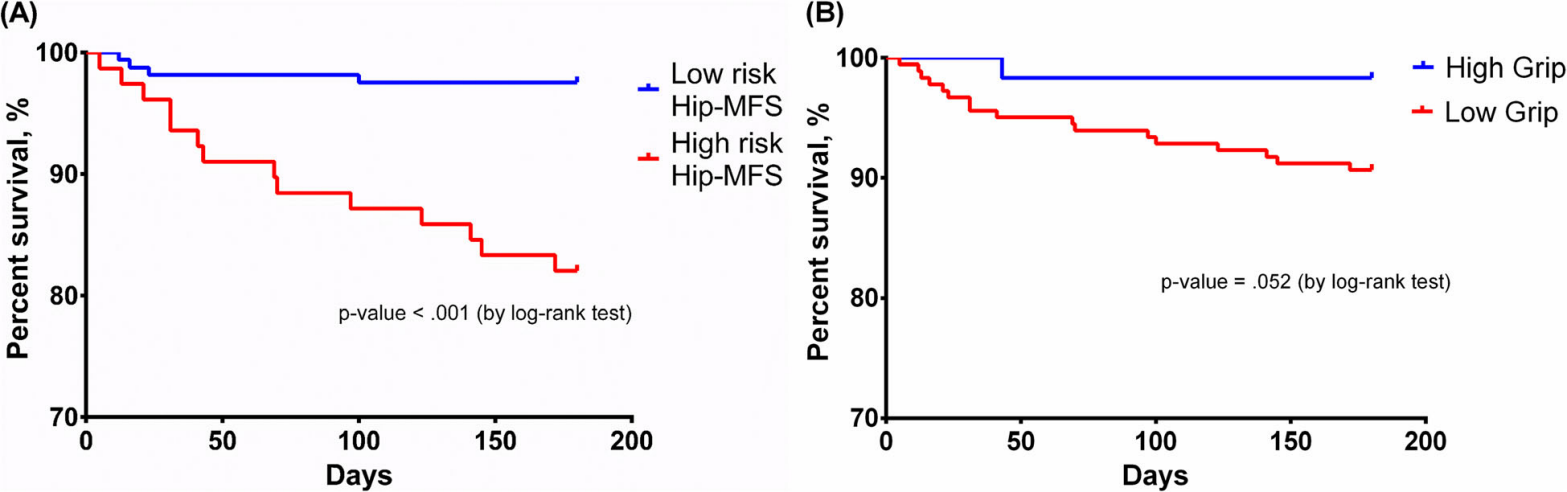
Cumulative 6-month mortality rate according to risk stratification based on the Hip-MFS and grip strength. Comparison of the Kaplan–Meier survival curves between (**a**) high-risk vs. low-risk Hip-MFS groups and (**b**) low vs. high grip strength groups. Log-rank test showing a significant difference between the Hip-MFS groups but not between the grip strength groups. Hip-MFS: hip-multidimensional frailty score

Similar trends were observed in the ORs and HRs in the models between the low HGS and high-risk Hip-MFS groups for predicting postoperative complications, 6-month mortality, and mortality at the end of follow-up (Supplementary Table 1). Furthermore, the cumulative 6-month survival rates of the low and high Hip-MFS risk groups were statistically significant according to the log-rank test of the Kaplan–Meier curve (*p* <  0.001), but not between the HGS risk groups (*p* = 0.052) **(**Fig. 3**).**

## Discussion

The Hip-MFS could predict both postoperative complications and mortality after HF surgery. HGS was a reliable predictor of complications but a weaker predictor of mortality than the Hip-MFS, while the ASA classification could only predict 6-month mortality. The results also confirmed that the evaluation of either the Hip-MFS or HGS, in addition to the ASA classification, improves the prediction of patients’ prognosis after HF surgery. The results of our investigation are consistent with those of previous research confirming the prognostic role of HGS or frailty after HF surgery. In previous studies, HGS could predict walking recovery, functional independence, or 1-year mortality [6, 19, 20]. Furthermore, frailty has predictive power for postoperative complications, increased length of inpatient stay, 30-day residence, and mortality [7, 21–23].

The Hip-MFS may be superior to HGS in prediction of adverse outcomes after HF surgery because it contains multiple domains obtained by CGA, while HGS only represents physical performance. Furthermore, the Hip-MFS is able to  represent the actual physiologic reserve or vulnerability, unlike HGS. Accurate stratification of decreased physiologic reserve and increased vulnerability to adverse outcomes might prompt clinicians to develop a better treatment strategy, enabling patients and providers to avoid, or at least to minimize, stressors and further risks.

The CGA could identify not only preoperative risks but also specific modifiable elements that should be intervened. A simple, feasible, and intuitive risk assessment tool could help surgeons develop a discharge plan or to decide whether co-management with a geriatrician is needed and whether there is a need to perform preemptive medical optimization, non-pharmacological delirium preventive intervention, and rehabilitation before or after an operation. However, the CGA has disadvantages in that it is labor-intensive and time-consuming. Thus, the results of this study suggest the need for implementing a two-step approach. Pre-screening patients at a high risk of postoperative complications, performing an HGS test, which is simple and can be performed at the bedside, and identifying patients who require further evaluation for frailty by performing the CGA. This two-step approach will help to lessen the use of medical resources and time compared to performing CGA in all patients.

A previous study comparing frailty and grip strength in older surgery recipients showed comparable results to this study [18]. Similar to a previous study, in this study, HGS alone did not predict short-term mortality after surgery. However, unlike in previous studies, the mortality at the end of follow-up could be predicted significantly in this study. These differences in results may be due to differences in age (mean age: 81.1 vs. 76.6), nutritional status (mean MNA score: 21.0 vs. 24.1), functional status (IADL dependency: 62.0% vs. 10.2%), and cognitive status (mean MMSE-KC score 18.0 vs 23.5) [18]. Furthermore, as the HF patients were older and frailer than the general surgical patient group, the majority of patients showed a phenotypical representation of frailty and decreased HGS. This finding suggests that the prognostic utility of HGS could be greater among older HF patients than among other general surgical populations.

Recently, many risk predictors of adverse outcome after HF have been identified and the importance of frailty or physical parameters such as HGS has been emphasized as a result [10, 24]. Though various risk score models have been developed, the prognostic utility of the Hip-MFS is superior to that of the most widely used Nottingham Hip Fracture Score [11]. To the best of our knowledge, this is the first study to compare the frailty score model from CGA with conventional anesthesia risk stratification tools, the ASA classification, and the emerging physical parameter, HGS. Our study also proves the additional prognostic utilities of the objective parameters, the Hip-MFS and HGS, compared to those of a more subjective tool, the ASA classification. In our study, low HGS had a higher prevalence (*n* = 182) than high-risk Hip-MFS (*n* = 78), and the majority (94.9%, *n* = 74) of high-risk Hip-MFS had low HGS. With additional ROC curve analysis, the cut-off value of HGS in predicting a high risk of frailty (Hip-MFS) was much lower than the cut-off value created for screening sarcopenia in the community population level. Analyzing by sex, an HGS cut-off value of ≤19.3 kg in men (sensitivity 84.2%, specificity 73.9%, A) and ≤ 12.7 kg in women (sensitivity 74.6%, specificity 73.7%, B) could predict a high risk of frailty (Supplementary Figure 1). Thus, we suggest that HGS could be used for screening, identifying frail patients, and targeting those who may benefit from the CGA under limited medical resources.

There are several limitations to this study. First, the included patients were retrospectively selected from a single center. Thus, further prospective studies including multiple institutions with a larger number of participants and longer observation periods are warranted. Second, the HGS measurement protocol did not adhere to the standardized method advocated by the American Society of Hand Therapists, in which the participants are tested in a seated position [25]. HGS was measured before surgery as its clinical implication seems to evaluate the postoperative risk. Although it is well known that muscle mass is maintained during the first 10 days after HF surgery, it is reasonable to measure HGS before surgery in a supine position. Indeed, this approach has also been used in other studies [26–28]. Third, even though the retrospective chart review to diagnose delirium was conducted by one geriatrician to minimize inter-observer variability, the incidence of delirium might be underdiagnosed due to hypoactive delirium or incomplete medical records. However, the incidence of delirium in this study (41.2%) was comparable with that in the previous study conducted in HF patients (28–50%) [29]. Fourth, 8.7% (*n* = 21) of HF patients experienced delirium before surgery, and given that the CGA was performed before surgery, the cognitive assessment could have been influenced by preoperative delirium. Though the decline in the cognitive function in the acute stage may be associated with frailty, we did not have a reliable assessment of premorbid cognitive function and adjust patients’ baseline cognitive function. When the Hip-MFS was calculated, the ADL/IADL was assessed based on function 1 month before fracture, and other objective indicators (laboratory finding, sex or mid-arm circumference) were also considered. Lastly, although our institution recommends the use of the CGA in older HF patients, it cannot be performed in all HF patients for reasons including patients’ refusal, failure to conduct the CGA as the patient is scheduled for surgery or other preoperative tests, and not requesting the CGA. However, in our previous studies conducted in our institution, no significant difference was observed in the baseline characteristics between participants and non-participants [11].

## Conclusion

The Hip-MFS showed better prognostic power for mortality after HF surgery than HGS. Both the Hip-MFS and HGS had incremental predictive ability for postoperative complications when added to the ASA classification. In conclusion, HGS is a reliable predictor of postoperative complications after HF and could be used for screening purposes to target frail patients who may benefit from further CGA.

## Supplementary Information

**Additional file 1: Supplementary Table 1.** Adjusted ORs and HRs by categorical cut-off values of grip strength and Hip-MFS for postoperative complication, 6-month mortality, and mortality at the end of follow-up. **Supplementary Figure 1**. Receiver-operating characteristic (ROC) curves based on grip strength to identify high-risk Hip-MFS in older male (A) and female (B) hip fracture patients.

## Data Availability

The datasets used and/or analyzed during the current study are available from the corresponding author on reasonable request.

## Abbreviations

ASA: American Society of Anesthesiologists
ADL: Activities of daily living
CGA: Comprehensive geriatric assessment
CI: Confidence interval
HGS: Handgrip strength
Hip-MFS: Hip-multidimensional frailty score
HR: Hazard ratio
IADL: Instrumental activities of daily living
ICU: Intensive care unit
IQR: Interquartile range
MMSE-KC: Korean version of the Mini-Mental State Examination
MNA: Mini Nutritional Assessment
Nu-DESC: Nursing Delirium Screening Scale
OR: Odds ratio
SD: Standard deviation
SGDS-K: Korea Geriatric Depression Scale
ROC: Receiver-operating characteristic
WBC: White blood cell
