# Supplementary material for: How does the multidimensional frailty score compare with grip strength for predicting outcomes after hip fracture surgery in older patients? A retrospective cohort study
Source: BMC Geriatr. 2021 Apr 7;21:234. doi: 10.1186/s12877-021-02150-9 (PMC8028224; doi:10.1186/s12877-021-02150-9)

**Supplementary Figure 1. Receiver-operating characteristic (ROC) curves based on grip strength to identify high-risk Hip-MFS in older male (A) and female (B) hip fracture patients.**


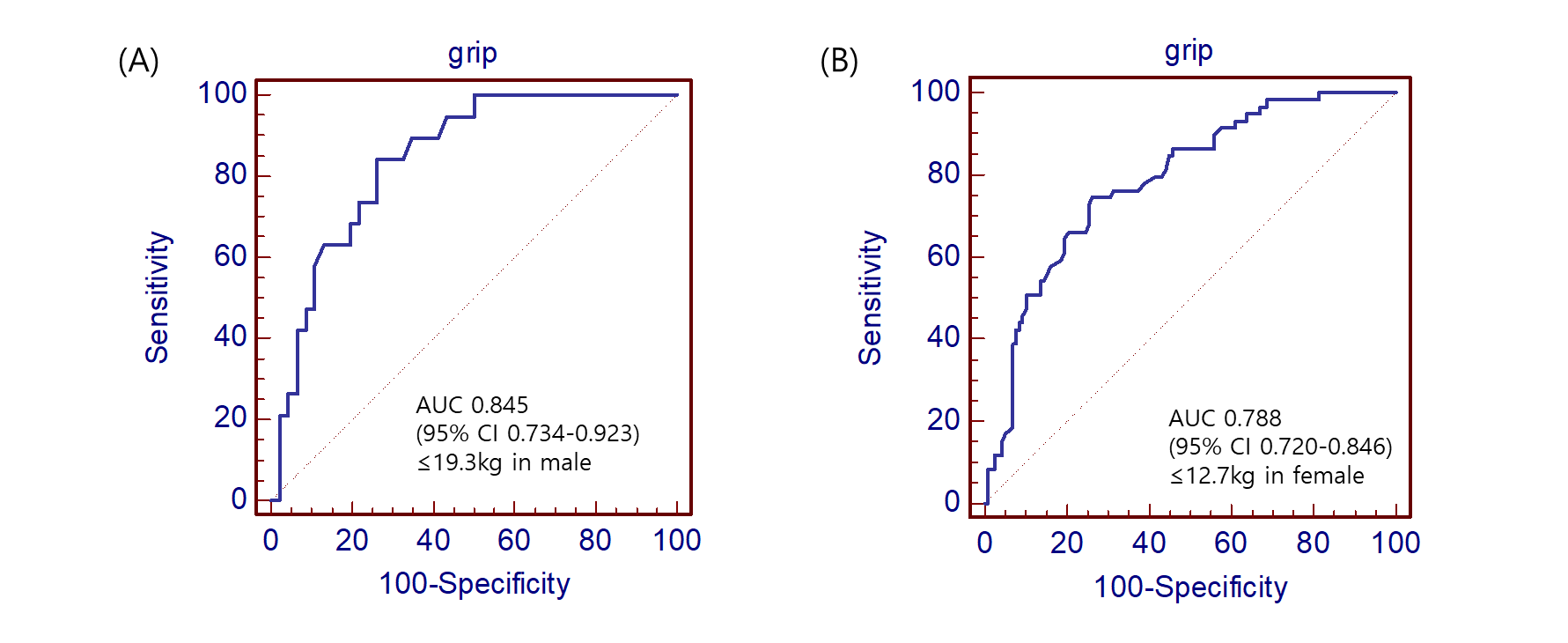

Supplement: Supplementary file 1 — Additional file 1: Supplementary Table 1. Adjusted ORs and HRs by categorical cut-off values of grip strength and Hip-MFS for postoperative complication, 6-month mortality, and mortality at the end of follow-up. Supplementary Figure 1. Receiver-operating characteristic (ROC) curves based on grip strength to identify high-risk Hip-MFS in older male (A) and female (B) hip fracture patients. [file 12877_2021_2150_MOESM1_ESM.zip › Supplementary Figure 1_ESM.docx]
