# Supplementary material for: How does the multidimensional frailty score compare with grip strength for predicting outcomes after hip fracture surgery in older patients? A retrospective cohort study
Source: BMC Geriatr. 2021 Apr 7;21:234. doi: 10.1186/s12877-021-02150-9 (PMC8028224; doi:10.1186/s12877-021-02150-9)
Supplement: Supplementary file 1 — Additional file 1: Supplementary Table 1. Adjusted ORs and HRs by categorical cut-off values of grip strength and Hip-MFS for postoperative complication, 6-month mortality, and mortality at the end of follow-up. Supplementary Figure 1. Receiver-operating characteristic (ROC) curves based on grip strength to identify high-risk Hip-MFS in older male (A) and female (B) hip fracture patients. [file 12877_2021_2150_MOESM1_ESM.zip › Supplementary_Table 1_ESM.docx]

**Supplementary Table 1. Adjusted ORs and HRs by categorical cut-off values of grip strength and Hip-MFS for postoperative complication, 6-month mortality, and mortality at the end of follow-up**

|  | **Model 1^a^** | **Model 2^b^** | **Model 3^c^** |
| --- | --- | --- | --- |
| **Complication (OR)** |  |  |  |
| High-risk Hip-MFS | 1.053 (1.010-1.097)^*^ | 2.097 (1.149–3.827)^*^ | 1.766 (0.925–3.371) |
| Low grip strength | 4.487 (2.084–9.661)^‡^ | 4.564 (2.115–9.847)^‡^ | 4.367 (1.985–9.611)^‡^ |
| **6-month mortality (HR)** | | | |
| High-risk Hip-MFS | 7.333 (2.295–23.430)^†^ | 6.083 (1.903–19.446)^†^ | 4.558 (1.103–18.830)^*^ |
| Low grip strength | 4.447 (0.568–34.801) | 4.018 (0.509–31.702) | 2.326 (0.280–19.360) |
| **Mortality at the end of follow-up (HR)** | | | |
| High-risk Hip-MFS | 3.827 (2.059–7.113)^‡^ | 3.695 (1.973–6.919)^‡^ | 2.725 (1.390–5.341)^†^ |
| Low grip strength | 2.545 (0.977–6.629) | 2.471 (0.947–6.444) | 1.824 (0.679–4.896) |

Data are presented as odds ratio or hazard ratio (95% confidence interval).

^a^ Adjusted by age, sex, and body mass index

^b^ Adjusted by age, sex, body mass index, and type of fracture

^c^ Adjusted by age, sex, body mass index, type of fracture, white blood cell count, hemoglobin, total cholesterol, protein, blood urea nitrogen, and creatinine

^*^; p < 0.05, ^†^; p < 0.01, ^‡^; p < 0.001
